# *In silico* analysis of the Seven IN Absentia (SINA) genes in bread wheat sheds light on their structure in plants

**DOI:** 10.1371/journal.pone.0295021

**Published:** 2023-12-21

**Authors:** Jane Roche, Claire Guérin, Céline Dupuits, Cherkaoui Elmodafar, Pascale Goupil, Said Mouzeyar

**Affiliations:** 1 UMR 1095 Génétique, Diversité et Ecophysiologie des Céréales, Université Clermont-Auvergne, INRAe, Clermont–Ferrand, France; 2 Faculté des Sciences et Techniques, Centre d’Agrobiotechnologie et Bioingénierie, Université Cadi Ayyad, Marrakech, Morocco; 3 UMR A547 Physiologie Intégrative de l’Arbre en environnement Fluctuant, Université Clermont-Auvergne, INRAe, Clermont–Ferrand, France; Government College University Faisalabad, PAKISTAN

## Abstract

Seven IN Absentia (SINA) is a small family of genes coding for ubiquitin-ligases that play major roles in regulating various plant growth and developmental processes, as well as in plant response to diverse biotic and abiotic stresses. Here, we studied the SINA genes family in bread wheat *Triticum aestivum* which is a culture of major importance for food security worldwide. One hundred and forty-one SINA family genes have been identified in bread wheat and showed that their number is very high compared to other plant species such as *A*. *thaliana* or rice. The expansion of this family seems to have been more important in monocots than in eudicots. In bread wheat, the chromosome 3 distal region is the site of a massive amplification of the SINA family, since we found that 83 of the 141 SINA genes are located on this chromosome in the Chinese Spring variety. This amplification probably occurred as a result of local duplications, followed by sequences divergence. The study was then extended to 4856 SINA proteins from 97 plant species. Phylogenetic and structural analyses identified a group of putative ancestral SINA proteins in plants containing a 58 aminoacid specific signature. Based on sequence homology and the research of that “Ancestral SINA motif” of 58 amino acids, a methodological process has been proposed and lead to the identification of functional SINA genes in a large family such as the *Triticae* that might be used for other species. Finally, tis paper gives a comprehensive overview of wheat gene family organization and functionalization taken the SINA genes as an example.

## Introduction

The regulation of gene expression in eukaryotic cells requires a wide variety of coordinated processes to ensure optimal cellular function. Transcriptional regulation is one of those processes, with, for example, the binding of transcription factors to specific regions of promoters. There are also post-transcriptional regulations, such as specific protein phosphorylation by protein kinases or targeted degradation of proteins by the Ubiquitin Proteasome 26S (UPS) pathway. This UPS pathway starts by the labeling of proteins to be degraded *via* an attachment of at least four ubiquitins. This ubiquitination process requires the sequential intervention of three enzymes: the ubiquitin activator E1, the E2 which conjugates the activated ubiquitin by transferring it to a third enzyme: the E3 ubiquitin-ligase. This last enzyme covalently attaches the ubiquitin to the target protein (substrate) *via* its lysine 48 (K48). After several repetitions of this cycle, the polyubiquitinated target protein is recognized and then degraded by the proteasome 26S. The E3 ubiquitin-ligase is the key enzyme that brings specificity towards the target protein. There are several families of E3 ubiquitin-ligases. Some of them are polymeric, with several subunits, such as the SCF complex [1]. Others are in a monomeric form carrying two properties: binding to the E2 enzyme and binding specifically to the target protein to ubiquitinate it. The Really Interesting New Gene (RING) is a class of monomeric E3 ubiquitin-ligases carrying a RING motif in their N-terminal region, and an interaction domain with the target protein in their C-terminal region. Classifications of E3 RING ligases have been proposed based on the nature of the RING motif but, within the same subgroup, the C-terminal domain varies according to the target proteins. Seven IN Absentia (SINA) is one of the RING subgroups carrying a SINA domain in their C-terminal region, which is involved in protein dimerization and interaction with the target protein [2,3]. SINA were first identified in *Drosophila melanogaster* [4].

In animals, the SINA proteins have been implicated in many key biological processes, *e*.*g*. apoptosis, autoimmunity, tumor suppression, response to hypoxia, leukemogenesis, etc. [5–9]. In a similar way, many SINA proteins have been described as playing a key role during plant development and response to biotic and abiotic stresses [10,11]. By interacting with ATG6, the SINAT1 and SINAT2 proteins described in *Arabidopsis thaliana* are involved in the autophagy pathway regulation [12]. In addition, the SINAT2 protein mediates carotenoid synthesis in leaves *via* its interaction with AtRAP2.2 [3]. Another E3 ligase, SINAT5, targets the transcription factor protein NAC1 and induces its degradation, affecting lateral root formation [13,14]. In rice, OsDIS1 gene expression is induced by drought and its silencing by RNA interference improves drought tolerance [15]. It has also recently demonstrated that SsSINA1 is involved in drought response in sugarcane [16]. Similarly, the MaSINA1 gene negatively regulates the cold response of banana by affecting the stability of MaICE1 [12]; while an overexpression of SlSINA4 in tomato activates cell death signaling [17]. In apple, MdSINA2 increases sensitivity to ABA [18]. In bread wheat, Thomelin et al. (2021) [19] recently described the E3 ligase TaSINA, which improves biomass and crop yield under heat stress. However, the SINA family as a whole has been little studied in plants. Li et al. (2020) identified 11 MdSINA genes in apple [18] while Wang et al. (2018) identified six members of this family in tomato, and found that each of them assembles with the others as homo- or heterodimers [17].

In this paper, we conducted an *in silico* analysis of the SINA gene family in bread wheat (*Triticum aestivum L*.*)*, an hexaploid species of major importance in human food security. We demonstrated that this family is widely expanded in bread wheat compared to other cereal species. A comprehensive survey of this family in various plant species and sequence comparisons was performed and lead to identify a likely ancestral subgroup. We also proposed a 58 amino acid (aa) motif as a signature of ancestral SINA sequences in plants.

## Results

### Identification of the SINA family members in bread wheat *Triticum aestivum*

The combined use of BlastP, using SINA sequences already characterized in *Arabidopsis thaliana* and *Solanum lycopersicum* as queries, and a manual curation based on the presence of the characteristic Interproscan motifs of SINA (N-ter RING motif and C-ter SINA motif) leads to the identification of 141 unique genes encoding SINA in the bread wheat *T*. *aestivum* var. Chinese Spring (CS) (**Table 1**). The length of TaSINA proteins varied from 134 to 1032 aa (TraesCS7D03G0166100.1 and TraesCS3D03G0031300.1 respectively), with an average of 334 aa. The number of SINA genes in the CS variety appeared larger than the SINA family identified in other plant species. In order to know whether the CS variety was an isolated case or whether this situation was general in bread wheat, we surveyed the number of SINA genes in 14 other sequenced bread wheat varieties (10 Genomes project [20]). The results displayed in **Table 1** supported the hypothesis of a particularly expanded SINA family not only in the CS variety but in the whole bread wheat group. Indeed, the SINA gene number varies from 141 genes in CS to 238 genes in Landmark and Stanley varieties, with an average of 197 genes per genome. This result confirmed that the number of SINA genes in bread wheat is close to 200 genes, which is a very large number compared with other species.

**Table 1:** Number of SINA genes identified in 15 bread wheat varieties.

Bread wheat variety	SINA gene number
**Chinese Spring**	141
ArinaLrFor	230
Cadenza	163
Claire	160
Weebill	162
Jagger	213
Julius	227
Lancer	219
Landmark	238
Mace	210
Norin61	231
Paragon	152
Robigus	163
Stanley	238
Sy Mattis	220

The genome of Chinese Spring used was extracted from [21]. The other genomes used were extracted from [20].

### SINA family expansion in the bread wheat genome

To know if the distribution of this high number of SINA genes was random, we then surveyed the *TaSINA* genes chromosomal anchorage. We observed that 58.8% of these genes (83 out of 141 in CS variety) are carried by chromosome 3, with 25, 33 and 25 *TaSINA* genes carried by chromosomes 3A, 3B and 3D respectively. Indeed, the chromosome 3B alone accounted for 23.4% of all the SINA genes in CS variety (S1 File). This result indicated that the family expansion did not occur randomly along the wheat genome but has mainly taken place on the chromosome 3. Moreover, it probably occurred before the hexaploidization of the wheat genome that gave rise to the current bread wheat. The same analysis about the distribution of SINA was performed in the 14 other bread wheat varieties (S1 File) and revealed a similar pattern: between 58 and 68% of the SINA genes identified in these varieties are anchored on chromosome 3.

### SINA anchorage on the chromosome 3 of the bread wheat genome

**Fig 1:**
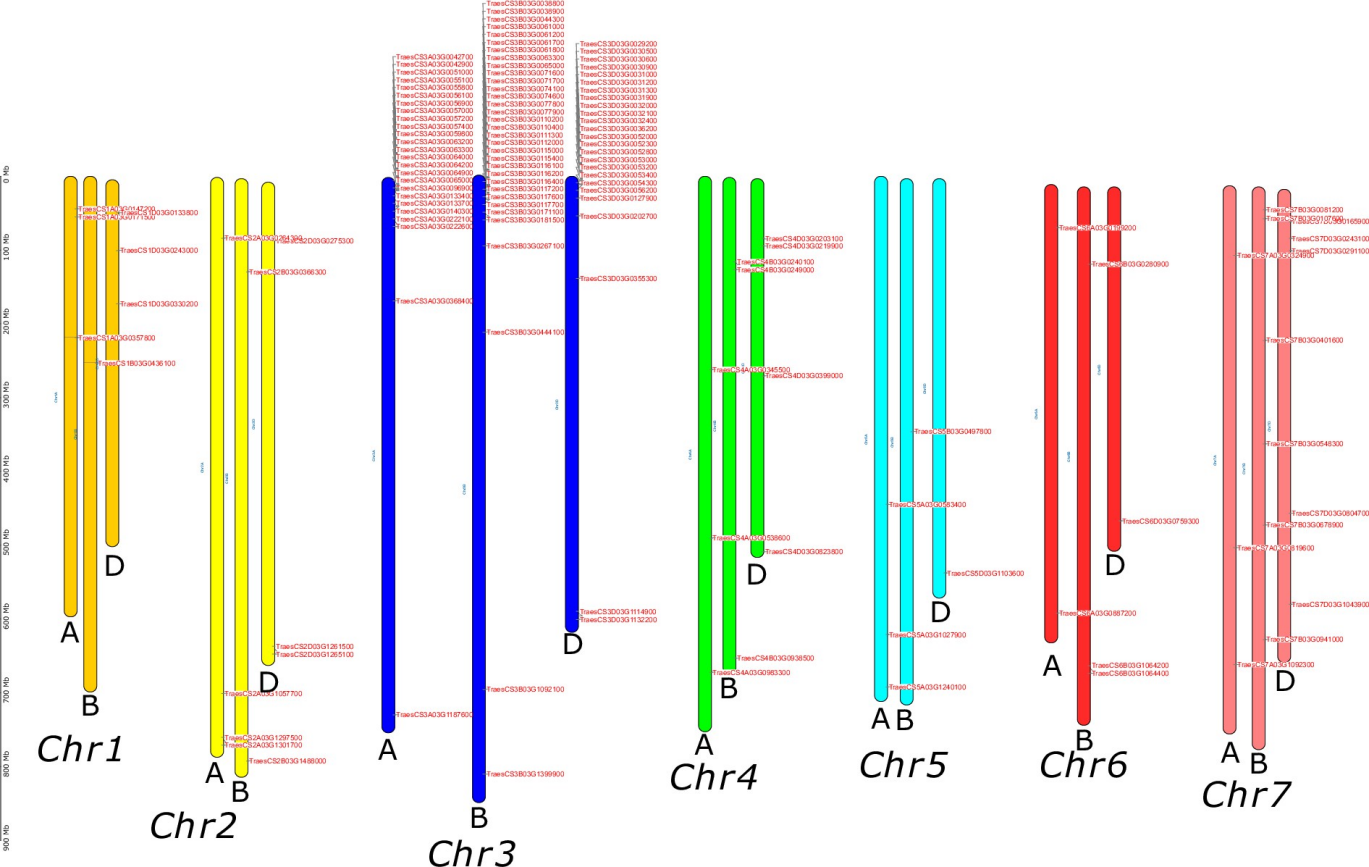
Anchorage of 141 SINA genes on the 21 bread wheat chromosomes of Chinese Spring variety. The three chromosomes represented with the same color are homoeologous chromosomes from A, B and D subgenomes. SINA genes are indicated in red.

The position of all the SINA genes in the CS variety was extracted and used to map the genes along the 21 wheat chromosomes. **Fig 1** clearly revealed a strong accumulation of the SINA genes at one end of chromosomes 3A, 3B and 3D. A fine analysis of the distribution of these genes along these chromosomes showed that they are regularly present as small clusters of two or more contiguous or tandem genes, sometimes separated by one or a few other genes. This distribution suggested that the amplification of the SINA genes family in bread wheat was probably achieved through local tandem duplications.

To go deeper into the evolution mechanisms of SINA genes, we focused on a particular region of the chromosome 3B containing 21% (7 out of 33) SINA genes anchored on this chromosome. This region of 1.26 Mb is localized between coordinates 32273198 and 33537271. We collected all the genes localized in this region yielding a set of 37 genes (ranging from TraesCS3B03G0115300LC.1 to TraesCS3B03G0119500LC.1). Among those 37 genes, 22 were annotated as High Confidence (HC) genes and 15 were annotated as Low Confidence (LC) genes by the IWGS Consortium (IWGSC). The latest are considered as hypothetical genes or pseudogenes by the IWGSC.

**Fig 2:**
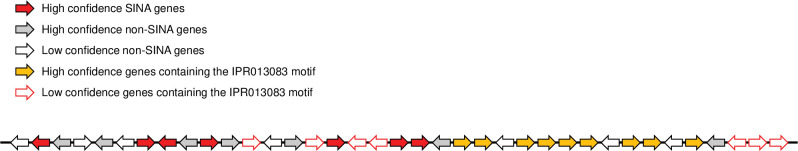
Chromosomal organization of the 37 genes anchored on the chromosome 3B of the bread wheat *Triticum aestivum* cv Chinese Spring. Each arrow represents one gene with its orientation in the pseudomolecule. IPR013083 motif is a Zinc finger, RING/FYVE/PHD-type/SIAH-type motif.

We studied their annotation and realized motif searches on the protein sequences encoded by these 37 genes. The results showed that 23 sequences encode for SINA-related proteins. Indeed, in addition to the seven High Confidence SINA sequences previously identified in this study (arrows filled in red in **Fig 2**), nine other HC sequences contained a “Zinc finger, RING/FYVE/PHD-type/SIAH-type motif” (IPR013083). Those nine genes (arrows filled in orange in **Fig 2**) can be considered as related to SINA genes, without being classified as true SINA genes. Similarly, among the 14 sequences identified as LC genes, seven are related to SINA genes (red empty arrows in **Fig 2**). As an example, it can be noticed that the six sequences TraesCS3B03G0118200.1, TraesCS3B03G0118300.1, TraesCS3B03G0118400.1, TraesCS3B03G0118500.1, TraesCS3B03G0118700.1, and TraesCS3B03G0118800. 1, anchored close to each other on the pseudomolecules, showed a very high identity rate. In fact, they differed only by one or two aa for most of them; or a deletion of 34 aa in TraesCS3B03G0118400.1 and another deletion of 20 aa in TraesCS3B03G0118200.1. Collectively, these results indicated that this region is particularly enriched in SINA-related sequences, suggesting a local duplication of those genes, some of which may have diverged and eventually degenerated into pseudogenes.

### Study of the SINA family expansion in the *Triticiae* subtribe

Because of the particularly high number of SINA genes in *Triticum aestivum*, we wanted to know whether it is also high in other *Triticum* species (S2 File). Therefore, we identified the SINA genes present in these species using the same approach as previously (based on the BLAST homology search and the presence of the SINA motif). For comparison’s sake, we analyzed this family in several non-Triticum plant species.

Overall, it appeared that the polyploidization phenomenon increased the absolute number of SINA genes. Indeed, if we take the example of the species *Camelina sativa* (*Brassicaceae*) which is an hexaploid species, the number of SINA identified is 80 genes, *i*.*e*. about 27 genes per diploid genome. This number is comparable to what was found in other diploid *Brassicaceae* such as *Arabidopsis thaliana* (20 genes) or *Arabidopsis halleri* (26 genes). In contrast, it was clear that the SINA gene family has been amplified very significantly in *Poaceae* in general and even more in the hexaploid bread wheat *Triticum aestivum*. Indeed, the Chinese Spring variety of this species contained 141 genes while *T*. *urartu*, one of the diploid ancestors of cultivated wheat, contained 41 genes. The high number in *T*. *aestivum* cannot be explained solely by the phenomenon of triplication of the genome (41 x 3 = 123 which is much lower than 141). It seems that the number of SINA genes is slightly amplified in diploid ancestors of wheat (*A*. *tauschii*: 39 genes, *T*. *urartu*: 41 genes) and that this expansion phenomenon is accentuated in hexaploid cultivated wheat. It is also important to note that this number varies very significantly between hexaploid wheat varieties. For instance, two varieties, Landmark and Stanley, contained each 238 SINA genes, which represented an increase of almost 69% compared to the Chinese Spring variety.

**Fig 3:**
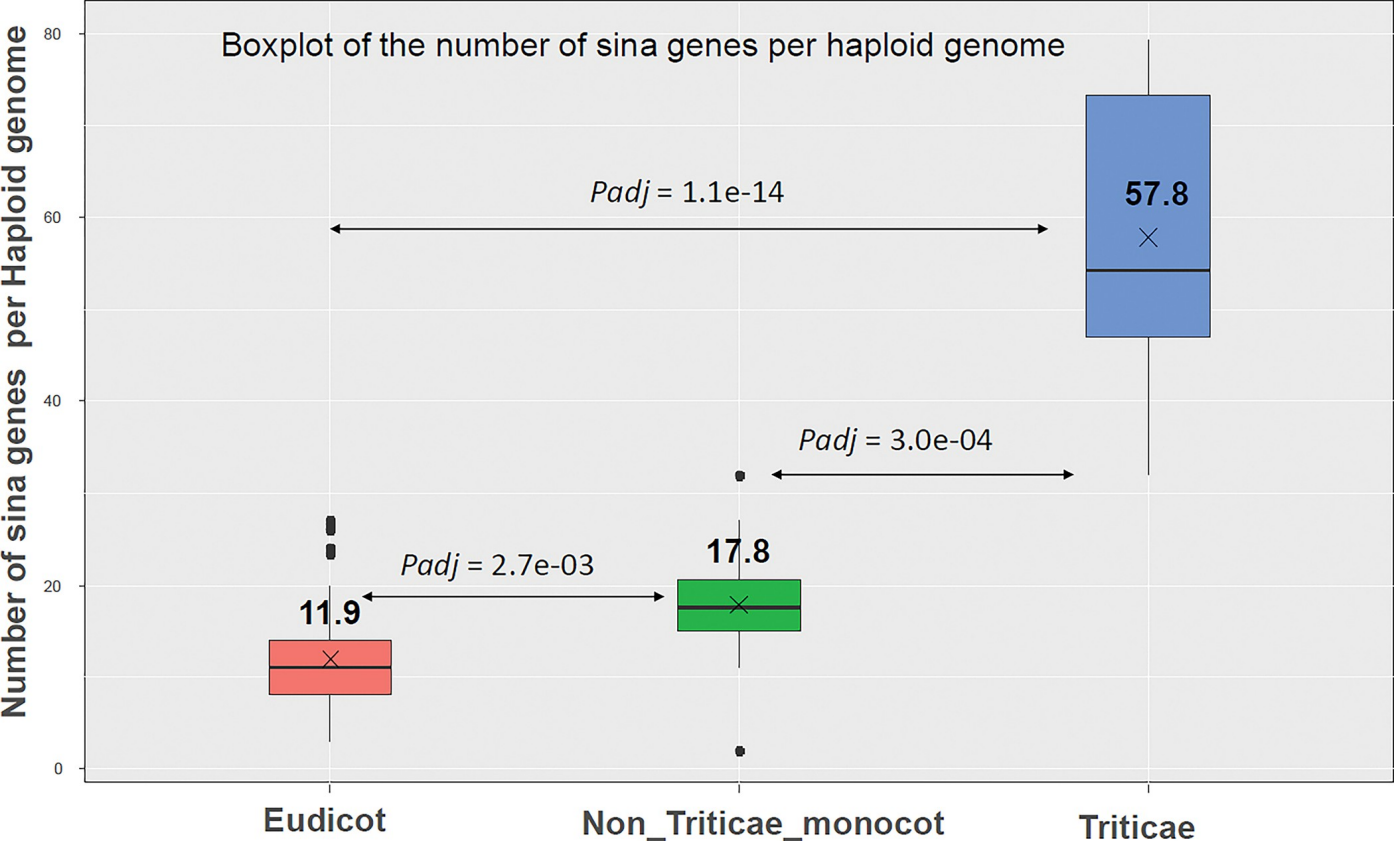
Boxplot of the number of SINA genes in 111 plant species which were categorized as Eudicots (n = 67), *Triticae* (n = 23) and non *triticae* monocot (n = 20). The mean number of SINA genes is given for each category. The adjusted pvalues for post-hoc Dunn Kruskal-Wallis test are given for each comparison (Benjamini-Hochberg correction).

We then compared the number of SINA genes per haploid genome in bread wheat and its relatives with other plant species. We grouped the 116 genomes (belonging to 97 species) into three groups: Eudicots (67 genomes), *Triticae* (*H*. *vulgare*, *T*. *dicoccoides*, *Aegilops tauschii*, *T*. *urartu*, *T*. *turgidum*, *Secale cereale*, 23 genomes) and the remaining non-triticae monocots (20 genomes). **Fig 3** shows a boxplot of the distribution of this number between these groups (see S2 File for the complete list). A Kruskal-Wallis test indicated that the average number of SINA genes is significantly different between these three groups. It is clear too that the SINA gene family has significantly amplified in monocots compared to eudicots, and this amplification was even more significant in the *Triticae*.

**Fig 4:**
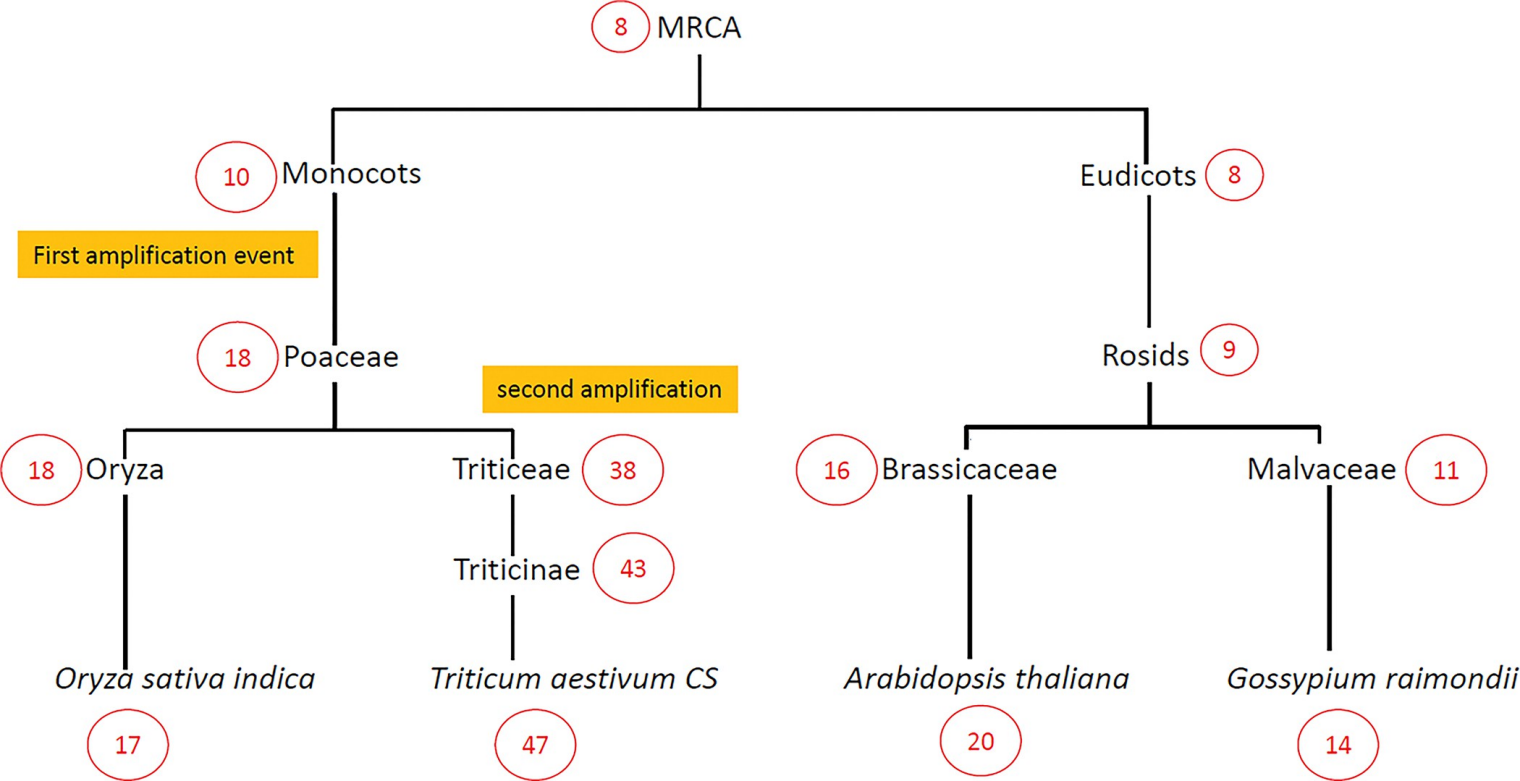
Evolutionary change of the number of SINA genes in plants. The Cladogram depicts a parsimonious reconstruction of the evolution of the number of SINA genes in plant kingdom (Cercled red text).

We then traced the evolutionary history of this gene family using the phylogenetic tree of *viridiplantae* species and the parsimony method to estimate the number of SINA genes in the MRCA (Most Recent Common Ancestor of the plant kingdom = *viridiplantae*) and different plant clades (**Fig 4**) and supplementary S3 File. This number was estimated at 8 in the MRCA, then 10 in monocots and 8 in eudicots. We then observed a first amplification event in the *Poaceae*, followed by a second amplification event during the separation between *Oryza* (18 genes) and the *Triticeae* (38 genes). Collectively, this information indicated that the SINA gene family has significantly amplified in the *Poaceae* compared to the rest of the plants. These observations also indicated that within the *Poaceae*, there was a second amplification event in the *Triticae*, and this amplification seemed to have continued in bread wheat *T*. *aestivum*.

### The expansion of the SINA family in the *Triticeae* was localized in chromosome 3

We then sought to describe the chromosomal distribution of SINA genes in different plant species. We chose the *Poaceae* family because it is the one for which we observed a very important expansion. **Table 2** showed the percentage of SINA genes contained in particular chromosomes of different species. Details of this count are given in S1 File. It is clear that within each botanical family, the expansion of the SINA family has taken place on only one or two particular chromosomes. In the *Triticinae*, the expansion has taken place mainly on chromosome 3, with percentages ranging from 51% to 68% of the whole set of SINA genes. In the *Oryzinae*, the expansion occurred on chromosome 1. In the *Panicoideae*, the expansion occurred on chromosomes 3 and 5. Since chromosome 1 in *Oryzinae* and chromosome 3 in *Triticinae* are known as being syntenic, these observations indicate that the expansion probably started in the common ancestor and must have continued in some current species. This is particularly plausible in the case of bread wheat, since it contained many more SINA genes than expected.

**Table 2:** Percentage of SINA genes contained in particular chromosomes of different species.

	Species	Percentage
**Chromosome 3 of *Triticinae***	*T*. *aestivum*	62%
	*T*. *spelta*	67%
	*H*. *vulgare*	59%
	*T*. *turgidum*	68%
	*T*. *dicoccoides*	61%
	*T*. *urartu*	56%
	*A*. *tauschii*	51%
**Chromosome 1 of *Oryzinae***	*Leersia perrieri*	59%
	*Oryza sativa Japonica Group*	47%
	*Oryza meridionalis*	63%
	*Oryza rufipogon*	63%
	*Oryza glumipatula*	55%
	*Oryza nivara*	55%
	*Oryza punctata*	50%
	*Oryza glaberrima*	40%
**Chromosome 3 of *Panicoideae***	*Sorghum bicolor*	56%
	*Panicum hallii FIL2*	36%
	*Setaria viridis*	27%
**Chromosome 5 of *Panicoideae***	*Panicum hallii FIL2*	48%
	*Setaria viridis*	40%
	*Panicum hallii*.*Phallii HAL*	55%

A high percentage of the SINA genes identified in *Triticinae* species are anchored on the chromosome 3. *Oryzinae* species harbor a high number of SINA genes on chromosome 1, which is known as syntenic of chromosome 3 in *Triticinae*.

### Phylogeny of SINA in plants

**Fig 5:**
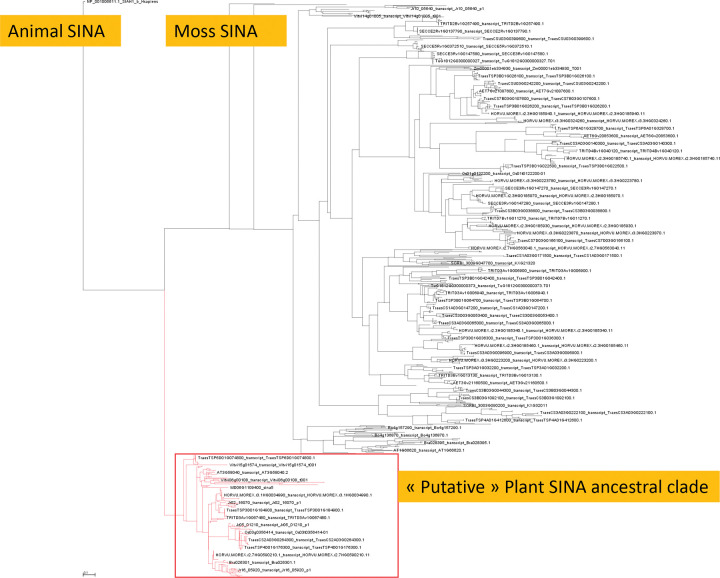
Rooted maximum-likelyhood tree from MAFFT alignment of 845 SINA proteins, from 25 species (23 *viridiplantae*, *Homo sapiens* and *Mus musculus*).

To better understand how SINA genes are structured in wheat, we conducted a phylogenetic analysis of SINA genes in a large panel of plant family. For this purpose, we collected SINA sequences from 25 species (23 plants species, *Homo sapiens*, and *Mus musculus*), for a total of 845 protein sequences. In terms of botanical classification, one *Bryophyta*, six Eudicots, 13 *Liliopsida*, and one *Lycopodiopsida* were used. ***Fig 5*** shows a rooted tree that includes several identifiable clades. In particular, one clade (colored in red) contained 178 sequences with at least one sequence belonging to one of the analyzed species, including the moss *P*. *patens* and the *Lycoppodiopsida S*. *moellondorfii* In addition, this clade showed phylogenetic proximity to sequences of animal origin (mouse and human) which were used as outgroups to root the tree. This suggested that this clade corresponded to highly conserved sequences between these species, and probably is the clade of ancestral sequences. In practice, sequences within this clade are closer to each other even if they come from phylogenetically distant species, compared to other sequences from the same species. Among the 178 sequences in this clade, we found the six well-characterized SINA genes in *A*. *thaliana* (AT2G41980.1_sina1, AT3G13672.2_sina6, AT3G58040.1_sina2, AT3G61790. 1_sina3, AT4G27880.1_sina4, AT5G53360.1_sina5), and 16 sequences from *T*. *aestivum* belonging to six homeologous groups (TraesCS1A03G0357800. 2, TraesCS1B03G0436100.1, TraesCS1D03G0330200.1, TraesCS2A03G0264300.1, TraesCS2B03G0366300.1, TraesCS2D03G0275300.1, TraesCS3A03G0368400.1, TraesCS3B03G0444100.1, TraesCS3D03G0355300.1, TraesCS4A03G0345500.1, TraesCS4D03G0399000.1, TraesCS6A03G0199200.1, TraesCS6B03G0280900.1, TraesCS7A03G0819600.1, TraesCS7B03G0678900.1, TraesCS7D03G0804700.1). These results suggested that bread wheat (CS variety) probably contains six ancestral SINA genes (belonging to six homeologous groups, 16 in total) and that probably these 16 genes would have been massively duplicated and then gradually diverged. We also identified putative ancestral SINA genes in the 22 other used plant species (S4 File for the complete table). We identified two other very large clades: the first included 59 sequences originating only from Eudicots and the second clade comprised 576 sequences originating only from *Poaceae*.

**Fig 6:**
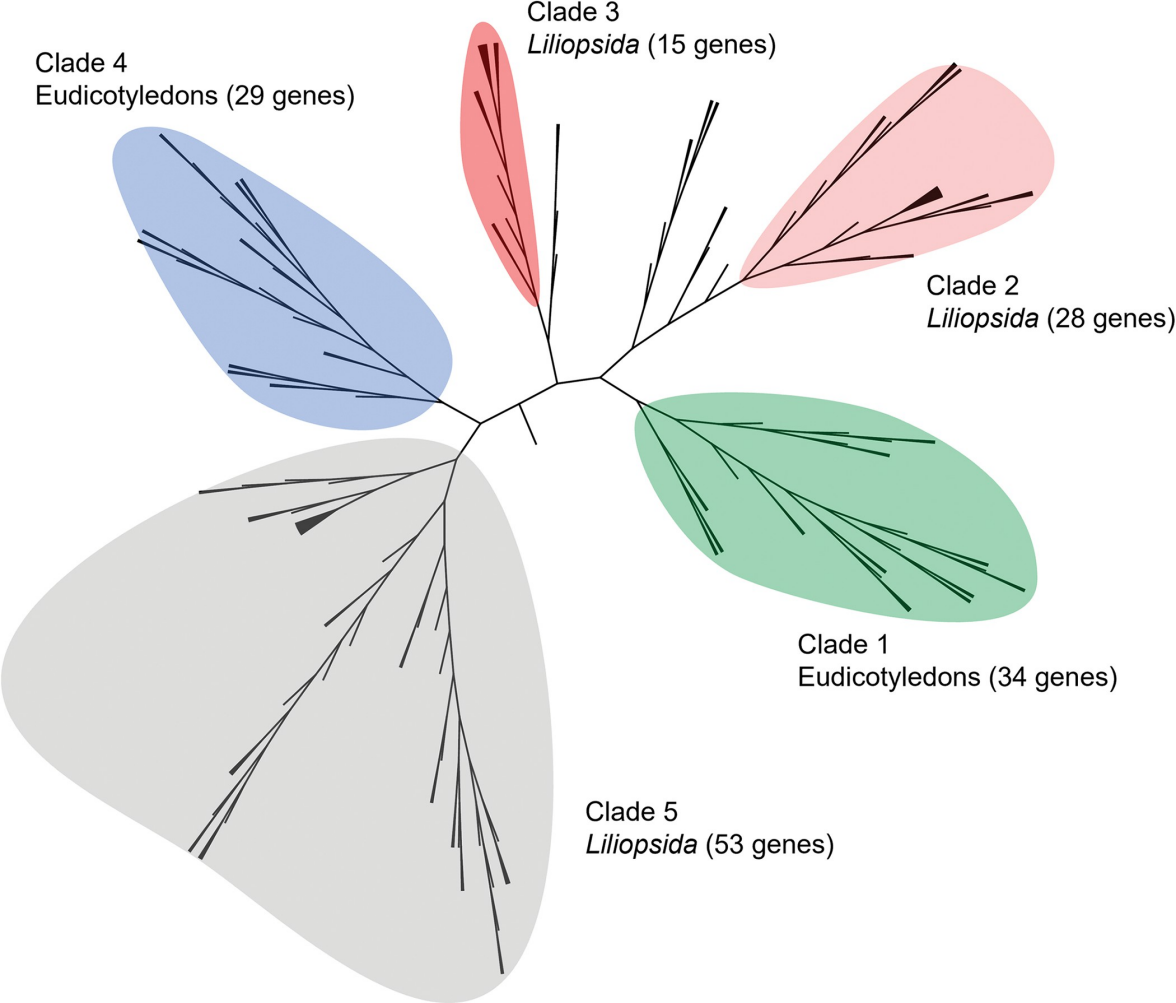
Zoom on the red clade of the tree described in Fig 5.

We then wanted to know if there was a structure within the clade of ancestor sequences. To do so, the subtree corresponding to this clade was isolated and expanded. As shown in **Fig 6**, there is a clear grouping of genes according to their botanical origin. We have identified five major clades. Clade#1 contained 34 genes including four genes from *Selaginella moellendorffii* (*Lycopodiopsida)*), two genes from *Physcomitrium patens* (Bryophyta, *Funariaceae*) and the remaining 28 genes from Eudicots such as *A*. *thaliana* (AT2G41980.1_sina1, AT3G13672.2_sina6, and AT3G58040.1_sina2). Clade#4 contained 29 genes exclusively from Eudicots, including *A*. *thaliana* (AT3G61790.1_sina3, AT4G27880.1_sina4, and AT5G53360.1_sina5). Clades#2, #3 and #5 contained 28, 15 and 53 genes, respectively. Clades#2 and #3 contained exclusively genes from *Poaceae*, while Clade#5 contained 52 genes from *Poaceae* and one gene from *Solanum lycopersicum* (*Solanaceae*, Eudicots). All these observations indicated that although these proteins showed a high level of identity, they must have diverged early enough in evolution to yield a clear distinction between Eudicots and *Liliopsida*. Nevertheless, if we cluster all the ancestral proteins using a global identity threshold of 65%, all the proteins are grouped in the same cluster except for the following sequences: NP_033198.1_SIAH1A_Mmusculus, XP_006530847.3_SIAH1A_X1_Mmusculus, Solyc12g100010.1, Vitvi15g01574, Bra003315, AET7Gv20281800 and Bra001519. Using a threshold of 80% identity, the sequences of Clades#4 and #5 are found in the same cluster. In particular, we found three SINA genes from *A*. *thaliana* (AT3G61790.1_sina3, AT4G27880.1_sina4 and AT5G53360.1_sina5) and nine sequences from *T*. *aestivum* (TraesCS1A03G0357800.2, TraesCS1B03G0436100.1, TraesCS1D03G0330200.1, TraesCS3A03G0368400.1, TraesCS3B03G0444100.1, TraesCS3D03G0355300.1, TraesCS7A03G0819600.1, TraesCS7B03G0678900.1 and TraesCS7D03G0804700.1). These nine sequences correspond to three homeologous groups on chromosomes 1, 3 and 7. These results suggested that these sequences in wheat are probably orthologs of the SINA3, SINA4 and SINA5 genes in *A*. *thaliana*.

### Identification of conserved motifs in SINA proteins

We then sought to further characterize the wheat SINA proteins by searching for the presence of specific motifs. To increase the relevance of motif identification, we used all proteins identified in *T*. *aestivum*, but also in *T*. *turgidum*, *T*. *dicoccoides*, and several well-characterized SINA sequences from *A*. *thaliana* (20 sequences), *H*. *sapiens* (2 sequences), *M*. *musculus* (2 sequences) *S*. *lycopersicum* (13 sequences), and *Malus domestica* (11 sequences). In total, 506 protein sequences were selected and submitted to MEME motif discovery tool [22]. The S5 File presents this set of 506 sequences with the distribution of the five motifs, and their logo representations. Motif#1 is 54 aa length, motif#2 is 56 aa length, motif#3 is 100 aa length, motif#4 is 55 aa and motif#5 is 53 aa length.

It is noteworthy that, among the 506 sequences analyzed, no protein sequence contained all the five motifs at the same time. Several different combinations of motifs were observed; however, we did not find any protein that included a combination of motif#3 with motif#4 or with motif#5. This suggests that motif#3 is exclusive of motif#4 and motif#5. The most common combination found was motif#2, motif#1, motif#4 and motif#5 (162 sequences), followed by the combination of motif#2, motif#1 and motif#4 (135 sequences) and finally 82 sequences that contained motif#3 distributed as follows: 72 contained motif#1, motif#2 and motif#3 whereas 10 sequences contained only motif#1 and motif#3.

In addition to the presence of different motifs, the sequences can vary by the presence of longer or shorter "stretches" without any detectable motif (S5 File). Some other protein sequences do not contain more than one motif among the five (*e*.*g*. TraesCS3A03G0222600.1 which contained only motif#1), or repeated motifs (*e*.*g*. TraesCS7D03G0166100.1 which contained twice motif#5). All these observations indicate that this family experienced dynamic sequence variations which led eventually to the acquisition/loss of rather broad domains which may certainly impact the proteins’ function.

**Fig 7:**
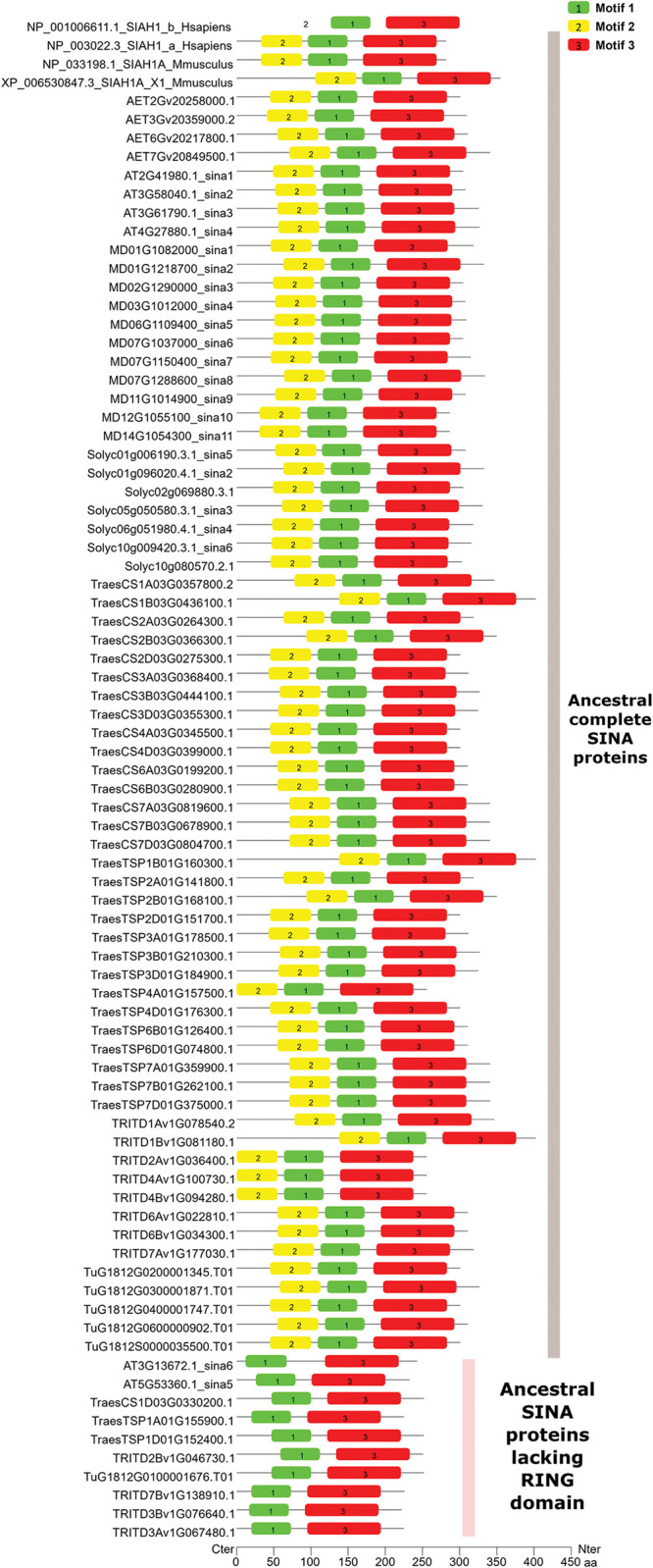
Motif discovery in 82 ancestral SINA sequences. Motif#3 associated with a RING domain would lead to identifying fully functional ancestral SINA proteins.

We then focused on the group of sequences that was qualified as ancestral in the phylogenetic analysis. **Fig 7** shows the presence and distribution of motifs in the ancestral sequences. Analysis of this figure indicates that all 82 identified sequences contained motif#1, motif#2 and motif#3, except 10 sequences which did not have motif#2. Note that among the 10 sequences lacking the motif#2, two corresponded to SINA5 and SINA6 in *Arabidopsis thaliana*, which are known to lack the RING motif depending on the ecotype. Collectively, phylogenetic analyses and motif searching suggested that the presence of motif#3 could be a hallmark of ancestral genes. Thus, we sought to further analyze this motif.

Motif#3, which is 100 aa lengths, was detected only in the 82 ancestral sequences among the set of 506 analyzed sequences. Alignment of the motif#3 extracted from these ancestral sequences revealed a highly conserved core sequence of 58 aa with some aa conserved at 100% and others aa less conserved. The following consensus sequence of 58 aa was then identified (with X representing any amino acid): HL2XD/HXV4XG2XFXHRYV9XAXWMLT3XCXG2XFXLXFEAFXL3XP. In the following text, we will call this core sequence "Ancestral SINA motif".

**Fig 8:**
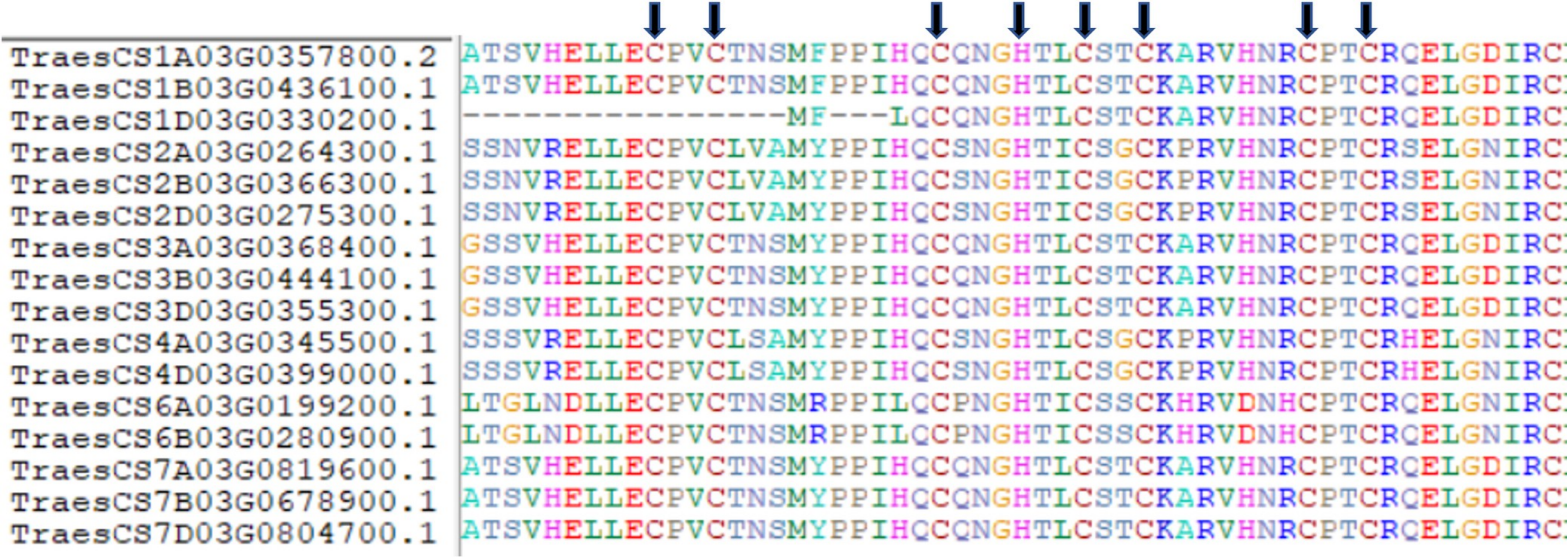
Sixteen SINA sequences containing the "Ancestral SINA motif". Black arrows represent the conserved cysteines and histidines belonging to the RING domain.

Most SINA proteins characterized so far contain a SINA domain that interacts with the target protein and a RING domain that leads to ubiquitination of this target protein. However, two SINA proteins were found in *A*. *thaliana* with a complete SINA domain but lacking a functional RING domain (AT5G53360.1_sina5, and AT3G13672.2_sina6). Physiologically, it has been shown that these two proteins can compete with SINA1/SINA2 to bind to the same target protein, protecting it from ubiquitination and thus from degradation [23]. In order to identify the complete set of ancestral proteins, we then used a two-step approach: firstly, by searching for the “Ancestral SINA motif” of 58 aa (derived from motif#3, see above); secondly, by searching for the presence of a canonical RING motif using the following regular expressions that identifies H2-type or HC-type of RING proteins: C[A-Z]{2}C[A-Z]{9,39}C[A-Z]{3,9}H[A-Z]{2,3}[C,H][A-Z]{2}C[A-Z]{4,48}C[A-Z]{2}C. Thus, in Chinese Spring, among the 141 SINA sequences identified in this study, 16 contained the "Ancestral SINA motif", 15 of which also contained a complete RING domain. The 16th sequence (TraesCS1D03G0330200.1) lacked the first two cysteines of the metal-ligand octet indicated by the arrows in **Fig 8**. This protein is certainly no longer capable of ubiquitination since the RING domain is not complete. It should also be noted that concerning the gene TraesCS1A03G0357800, its isoform#2 (TraesCS1A03G0357800.2) was retained as complete, whereas its isoform#1 had a very large deletion that eliminated the whole RING domain. So, the Chinese Spring variety presents 14 complete ancestral SINA sequences and two ancestral SINA sequences lacking a RING domain. The remaining sequences showed a highly rearranged SINA domain. For comparison, the same analysis showed that there are 20 SINA sequences in *A*. *thaliana*, of which six are ancestral (four complete and two with non-functional RING domain).

This approach was then applied to a very large panel of 97 plant species and corresponding to 115 varieties/ecotypes. Four categories of SINA genes were defined: SINA genes with ancestral SINA motif and RING motif (“Ancestral SINA motif and RING domain”), SINA genes with only the ancestral SINA motif (“Ancestral SINA motif without RING domain”), SINA genes with a rearranged SINA motif and a RING motif (“Modified SINA motif and RING domain”) and finally SINA genes with the rearranged SINA motif and without RING domain (“Modified SINA motif without RING domain”). All these results are presented in S6 File. **Table 3** shows a survey in 14 cereals. It can be clearly noticed that the distribution varies between varieties (cultivars) from the same species. However, the number of complete ancestral SINA genes (with Ancestral SINA motif and RING domain) seems to vary less than the other classes. This is probably due to the fact that this class of protein is involved in conserved essential physiological functions and is therefore less likely to evolve. Note that not all species/varieties necessarily contained a representative of the 2nd class (Ancestral SINA motif without RING domain).

**Table 3:** Classification of SINA proteins of eight plant species according to the presence of a SINA motif (ancestral or modified) and a RING domain.

	Ancestral SINA motif and RING domain	Ancestral SINA motif without RING domain	Modified SINA motif and RING domain	Modified SINA motif without RING domain	Total per genome
***Triticum aestivum* Arinalrfor**	16	0	128	86	230
***Triticum aestivum* Cadenza**	14	2	100	47	163
***Triticum aestivum* Chinese Spring**	15	1	86	39	141
***Triticum aestivum* Jagger**	15	2	114	82	213
***Triticum aestivum* Julius**	16	1	119	91	227
***Triticum aestivum* Lancer**	17	0	120	82	219
***Triticum dicoccoides***	11	2	39	17	69
***Triticum turgidum***	11	1	53	29	94
***Triticum urartu***	5	1	24	11	41
***Aegilops tauschii***	5	0	19	14	38
***Oryza sativa Japonica***	6	0	5	4	15
***Oryza sativa Indica***	5	0	7	5	17
***Oryza brachyantha***	6	5	11	1	23
***Arabidopsis thaliana***	4	2	10	4	20

Finally, it is also interesting to note that, whatever the variety, the number of SINA genes in “Ancestral SINA motif and RING domain” class is always greater than that of “Ancestral SINA motif without RING domain” class. This result suggests that those last genes are likely derived from the “Ancestral SINA motif and RING domain” class after loss of the RING domain due to mutations. For example, in Chinese Spring, the TraesCS1D03G0330200.1 gene has lost the first two cysteines of the metal-ligand octet indicated by the arrows and compulsory for a functional RING domain (**Fig 8**). If all the data from 97 plant species analyzed in this study are aggregated, a total of 4856 SINA genes is found (**Table 4**).

**Table 4:** Number of SINA genes identified in 97 plant species, classified according to the presence of a SINA motif (ancestral or modified) and a RING domain.

	Number of genes
**Ancestral SINA motif and RING domain**	784
**Ancestral SINA motif without RING domain**	72
**Modified SINA motif and RING domain**	2553
**Modified SINA motif without RING domain**	1447
**TOTAL**	4856

The complete table as well as the sequences are given in S4 File. At least one sequence containing the two motifs “Ancestral SINA motif” and “RING domain” was identified in representatives’ genomes of all plant species: Eudicotyledons, *Liliopsida*, *Amborellales* (*Amborella trichopoda*), *Embryophyta* (*Papaver somniferum*, *Nymphaea colorata*, *Physcomitrium patens*, *Marchantia polymorpha*), *Streptophyta* (*Chara braunii*) and *Lycopodiophyta* (*Selaginella moellendorffii*).

## Discussion

This work sought to characterize the SINA gene family in the hexaploid cultivated bread wheat *Triticum aestivum*, variety Chinese Spring. Our results showed that this family is relatively large compared to what has been observed in other plant species such as *A*. *thaliana*. For comparison’s sake, the analysis was then extended to 97 plant species, covering all major botanical groups and whose genome is sequenced. When possible, different varieties/cultivars or ecotypes belonging to the same species were included.

### Methodological choices

The functional SINA genes characterized so far contained a C-terminal SINA domain involved in the interaction with the target protein and an N-terminal RING domain that is responsible for the ubiquitination of the target protein. We exploited these features to identify SINA genes in wheat. In particular, we started by selecting all proteins that have a SINA domain and then characterized these proteins. We decided to be very stringent by using only the Interproscan motifs IPR018121, IPR004162 and IPR044286. As these proteins are a subclass of the large family of proteins containing a TRAF (Tumor necrosis factor Receptor-Associated Factor) domain [24], they may show homologies with other TRAF proteins in BLAST-based research strategies. Therefore, proteins containing the IPR008974 (TRAF) motif were excluded from our analysis. In addition, mRNAs from eukaryotic genes can undergo alternative splicing generating different isoforms. For this reason, during the SINA gene retrieving step, all transcripts of each gene were screened and the isoform containing the maximum of Interproscan motifs was retained.

### The SINA gene family has been expanded in *Triticeae* specifically in chromosome 3

When comparing the number of SINA genes in wheat and other plant species, we observed that wheat has an average of 197 SINA genes, ranging from 141 genes in the Chinese Spring variety to 238 genes in Landmark and Sy Mattis varieties. This result has to be compared with the 20, 17 and 15 SINA genes found in *A*. *thaliana*, *Oryza sativa Indica* and *Oryza sativa Japonica* respectively. This difference cannot be explained only by the wheat hexaploidy and this situation is not exclusive to this gene family. Indeed, several studies have shown that there is an expansion of certain gene families in wheat. For example, the MAD-box and NAC families of transcription factors contain 201 and 488 genes respectively, which have probably expanded in wheat through tandem duplication, retrotransposition and small-scale duplications [25,26]. The same observation has been made for the F-box family, which include 3670 members in bread wheat [27].

Similarly, our results showed an expansion of this family in other *Triticum* species, especially the diploid and tetraploid ancestors. Chromosomal localization of this family members showed that chromosome 3 alone comprised between 51% (*T*. *urartu*) and 68% (*T*. *turgidum*) of the total SINA genes identified in each genome. In *T*. *aestivum*, chromosome 3 (3A, 3B, and 3D) contained 62% of the SINA genes, which is far from the 14% expected if the distribution was random among the 21 chromosomes. This probably reflects a predominance of localized short duplications as a mechanism of expansion of this family. A possible scenario suggests that once the first localized duplications have occurred at a locus, new local duplications will occur from the newly duplicated genes. This process may lead to a local exponential process. This scenario has already been proposed for other gene families such as the one coding for aci-reductone dioxygenase-like proteins. For this family, the authors have shown that the ancestral gene is found on chromosome 1 while the duplicated copies are found on chromosomes 2, 3, and 4 and that the newly duplicated genes give rise to new copies in turn [28]. It is worthy to mention that a similar phenomenon is observed in the chromosome 1 of *Oryzinae*, which contains between 40% and 63% of the SINA genes depending on the species. This is also consistent with the fact that chromosome 3 in *Triticum* is syntenic with chromosome 1 in rice [29,30].This suggests that the first duplications would have occurred in the common ancestor of *Triticinae* and *Oryzinae* and that this would have continued more actively in *Triticinae* in general and in *T*. *aestivum* in particular. Interestingly, these local duplications have primarily occurred in the distal part of chromosome 3, called R1 according to IWGSC [21] and where recombination frequency is higher than in other so-called "proximal" regions. This has also been observed for other gene families that have expanded such as genes encoding MADS-Box transcription factors [25,31]. This process may lead to the creation of notable variations that result in the appearance/deletion of domains with the consequence that each locus where these duplications occur becomes a potential reservoir for multiple rounds of genes birth and death.

We also observed that the number of SINA genes varied between varieties within bread wheat species, ranging from 141 in Chinese Spring to 238 in Landmark or Sy Mattis varieties. This is in agreement with the results described by Walkowiak et al. (2020) who sequenced 15 wheat varieties and analyzed the nucleotide-binding leucine-rich repeat (NLR) class of resistance genes [20]. They showed that the number of NLR genes varied from 2326 genes in the variety Weebill1 to 2701 genes in the variety Norin61. These authors showed that about 26% of the genes are found in regions of tandem duplications, which would indicate that copy number variation contributed significantly to genetic variation between wheat varieties.

Similarly, Hao et al. (2020) sequenced 145 wheat varieties in China and showed that modern Chinese wheat varieties had higher genetic diversity than older ones (landraces) [32]. These authors suggested that this variation might be related to recent breeding programs applied by breeders to select agronomic traits.

### Identification of ancestral SINA genes in plants in general focus on wheat

A phylogenetic approach was combined with a structural analysis of SINA proteins in 97 plant species corresponding to 115 different varieties/ecotypes. A class of SINA proteins was then identified and considered as ancestral, because they form a distinct clade which contained at least one gene from each of the 97 plant species analyzed. This may suggest that these proteins are involved in ancestral conserved functions in plants. The number of ancestral SINA proteins per species is relatively low, even in species where this gene family has expanded. Indeed, in bread wheat, this class included only around 15 genes (depending on the variety) while the whole family contains up to 238 members. These ancestral proteins are characterized by the presence of two domains: a SINA domain and a RING domain. However, SNPs or short deletions/insertions may sometimes modify the RING domain and probably lead to a non-functional RING domain. Indeed, in Arabidopsis, SINAT5 (AT5G53360) contains a functional RING domain in the Landsberg ecotype but not in the Columbia ecotype [33]. Similarly, SINAT6 in *A*. *thaliana* (AT3G13672) contains a SINA domain but no RING domain and participates in the control of autophagy *via* the protection of the ATG13 protein. Indeed, under rich nutrition conditions, SINAT1 (At2g41980) and SINAT2 (At3g58040) associate with TRAF1a/b and lead to the ubiquitination of ATG13 and its degradation by the 26S proteasome, thus preventing the autophagosome from assembling. In contrast, under carbon starvation, SINAT6 binds to ATG13 without causing its ubiquitination and degradation, and the autophagosome can then be assembled [34]. This example shows that, although a SINA protein does not possess all functional domains (RING domain in particular), it can have an important physiological role *via* its interaction with other proteins. In bread wheat, some varieties contained one or two ancestral SINA genes lacking a functional RING domain, while in other varieties, no such gene has been identified (ArinaLrFor and Lancer varieties). In general, very few ancestral SINA genes with a non-functional RING domain were identified in all the analyzed species. For instance, over the 4856 SINA genes identified, only 72 belong to this category. This low number probably reflects a subtle and rare adaptation that may exist in some varieties but not in others, as it is the case in *A*. *thaliana* where the Landsberg ecotype contains SINAT5 (AT5G53360) with a functional RING domain but not Columbia variety [33].

However, this repertoire of ancestral SINA genes with functional or non-functional RING domain is based only on *in silico* analysis of protein sequences and the presence of canonical motifs. It should therefore be verified experimentally for each species by conducting *in vitro* autoubiquitination reactions for example.

In addition to the ancestral SINA genes, we have identified genes coding for proteins that contained either a highly-modified SINA domain (often shorter than the canonical SINA motif), or a modified or even completely absent RING domain. This is probably due to a phenomenon of pseudogenization which would have taken place following duplication processes [30,35]. Nevertheless, this very large pool of apparently non-functional proteins may be a source of adaptation. Indeed, it is possible that a SINA protein could no longer ubiquitinate its target, but still retains the ability to physically bind it. By this mechanism, the SINA protein can contribute to the protection of the target protein from degradation by the 26S proteasome, as for the case of SINAT6 (AT3G13672) in *A*. *thaliana* which binds and protects the ATG13 protein during autophagy control [23]. Recently, Thomelin et al. (2021) found a very strong association between a polymorphism in a TaSINA gene promoter (TraesCS3B02G572900) and a QTL controlling several yield parameters (early vigor, seminal root number, plant height, spike length, above-ground biomass, stem and spike biomass, spike number, number of spikelets per spike, and total grain number in deep soil following drought and heat stress). They then proposed a model in which overexpression of this gene would promote ubiquitination and degradation of a positive growth regulator. However, analysis of the structure of this protein showed that it probably lacks ubiquitination capacity and that it may be involved through the protection of a negative growth regulator in wheat [19].

### Identification of a signature in ancestral SINA genes

**Fig 9:**
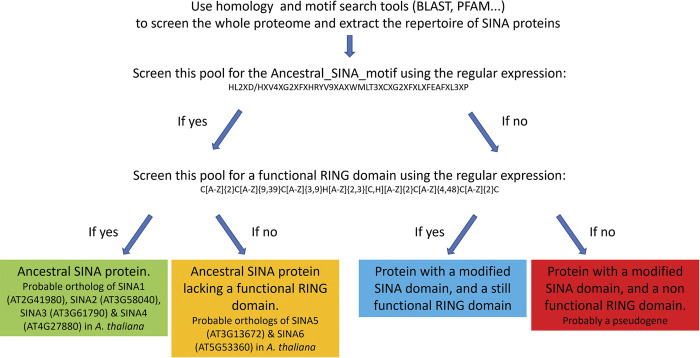
Methodological process proposed for the identification and classification of the SINA proteins in plant species.

By comparing a total of 4856 SINA sequences, a characteristic sequence of ancestral SINA proteins, was identified. This signature sequence is 58 aa long (HL2XD/HXV4XG2XFXHRYV9XAXWMLT3XCXG2XFXLXFEAFXL3XP where X represents any aa) and was named "Ancestral SINA motif". We propose the following hypothesis: ancestral SINA proteins involved in conserved metabolic pathways, such as autophagy, necessarily contain this motif and a (very often functional) RING domain. Point mutations and/or larger or smaller deletions/insertions may lead to sequence variations of these domains. Of course, these domains can still be detected by tools such as PFAM or InterProScan which tolerate mismatches and allow the detection of somehow distant homologies [36]. Therefore, with homology and domain search tools, it is possible to detect a RING domain without the eight canonical "metal-ligand" amino acids necessary for its functioning. Similarly, it is possible to detect a SINA domain that may be highly rearranged with respect to a canonical SINA domain, with a lower but nonetheless significant associated p-value. For these reasons, we propose the following approach to characterize all SINA proteins in a plant species (**Fig 9**) (see S4 File in complement). It should be noted that the methodological approach described here is not valid for non-plant species.

## Conclusion

Using comprehensive approaches, such as homology search, sequence comparison, phylogeny, and protein structure analysis combined with manual curation, we characterized the SINA gene family in wheat. This analysis was then extended to all cereals and to a very large panel, representative of all plant botanical groups. We subsequently proposed a procedure to explore this family of genes in any plant species in order to identify candidate orthologs of known genes in model plants such as *Arabidopsis thaliana*. With the increasing availability of sequenced genotypes/ecotypes in plant species of agronomic interest, this approach could be also incremented at the subspecies/variety level.

## Materials and methods

Sequences of the genomes used in this study were downloaded from https://plants.ensembl.org on 04/16/2022. The species names and the genomes versions are given in S7 File.

Triticum_aestivum_iwgsc_refseqv2.1_gene_annotation_200916.fa stands for the reference genome of Chinese Spring according to the International Wheat Genome Sequencing Consortium [21]. For the other genomes, references are indicated in Ensembl Plants’ website. For each genome, all gene versions (isoforms) were screened and for each gene, only the version which complied with the screening criteria was retained for the rest of the analysis. Protein sequences which do not start with Methionine and/or contain unknown aa (x) were excluded.

Sequence manipulations and analysis were carried out using TBtools suite version v1.09876 and tools therein [37].

### Identification of SINA proteins in wheat

Well characterized SINA proteins from *A*. *thaliana* (AT2G41980.1_sina1, AT3G58040.1_sina2, AT3G61790.1_sina3, AT4G27880.1_sina4, AT5G53360.1_sina5, AT3G13672.2_sina6) and from apple [18] were used as queries in BlastP searches. Positive hits in wheat proteome were then examined for the presence of characteristic SINA domains using Interproscan and the motifs IPR018121, IPR004162 and IPR044286. Finally, this set was manually checked and those sequences containing inconsistent structures were excluded.

### Localization of SINA genes on the chromosomes of wheat

The genomic coordinates of wheat SINA genes on the wheat genome pseudomolecule were used to analyze the chromosome distribution of this family [21]. Chromosome representations were done using the Tbtools suite [37].

### Motif discovery in protein sequences

MEME motif discovery tool version 5.0.5. was used to identify conserved motifs within the protein sequences. After initial testing of several parameters, the following parameters were selected: mod = any number of repeats, number of motifs = 5, e-value threshold = 1e-10, min width = 40, and max width = 120 [22].

### Sequence alignment and phylogeny analysis

Sequences were first aligned using MAFFT with default settings [38]. The alignment was then trimmed to remove all large unaligned regions using TrimAL with ML_AUTOMATED1 trimming mode [39]. The trimmed alignment was then used to construct Approximately Maximum-Likelihood trees using FastTree Version 2.1.10 [40] with the following parameters: Amino acid distances: BLOSUM45, Joins: balanced, Support: SH-like 1000, Search: Normal +NNI +SPR (2 rounds range 10) +ML-NNI opt-each = 1, TopHits: 1.00*sqrtN close = default refresh = 0.80, ML Model: Jones-Taylor-Thorton, CAT approximation with 20 rate categories [40]. Trees were rendered using Archaeopteryx version 0.9928beta (https://sites.google.com/site/cmzmasek/christian-zmasek/software/archaeopteryx, consulted the 30^th^ of March 2021.

### Inference of the evolution of the number of SINA genes

To analyze the expansion of the SINA family, we computed the number of SINA genes in each modern plant species per haploid genome. Briefly, the number was divided by 3 in hexaploid species, by 2 in tetraploid species and kept as it is for diploid and haploid species. By doing so, the effect of the polyploidization was neutralized. Ancestral gene count reconstruction was performed using Mesquite software (https://www.mesquiteproject.org/, version 3.81 april 2023) based on *Viridiplantae* species phylogenetic tree. The analysis utilized the parsimony method to infer the most likely number of genes at ancestral nodes.

## Supporting information

S1 File Number of SINA genes per chromosome in wheat varieties and other cereals. (XLSX)

S2 File Number of SINA genes in each class in selected plant genomes. (XLSX)

S3 File Number of genes under a parsimony reconstruction. (PDF)

S4 File Summary of classification and characteristics of 4856 SINA genes in plants. (XLSX)

S5 File Motifs detection in 506 SINA genes with logos. (PDF)

S6 File Number of SINA genes in each class in total plant genomes. (XLSX)

S7 File List of genomes used in this study. (XLSX)
