# Supplementary figures and images for: In silico analysis of the Seven IN Absentia (SINA) genes in bread wheat sheds light on their structure in plants
Source: PLoS One. 2023 Dec 21;18(12):e0295021. doi: 10.1371/journal.pone.0295021 (PMC10734943; doi:10.1371/journal.pone.0295021)

Character 1:  
nombre\_de\_genes  
Parsimony reconstruction  
(Squared) [Squared  
length: 1863.95103317]

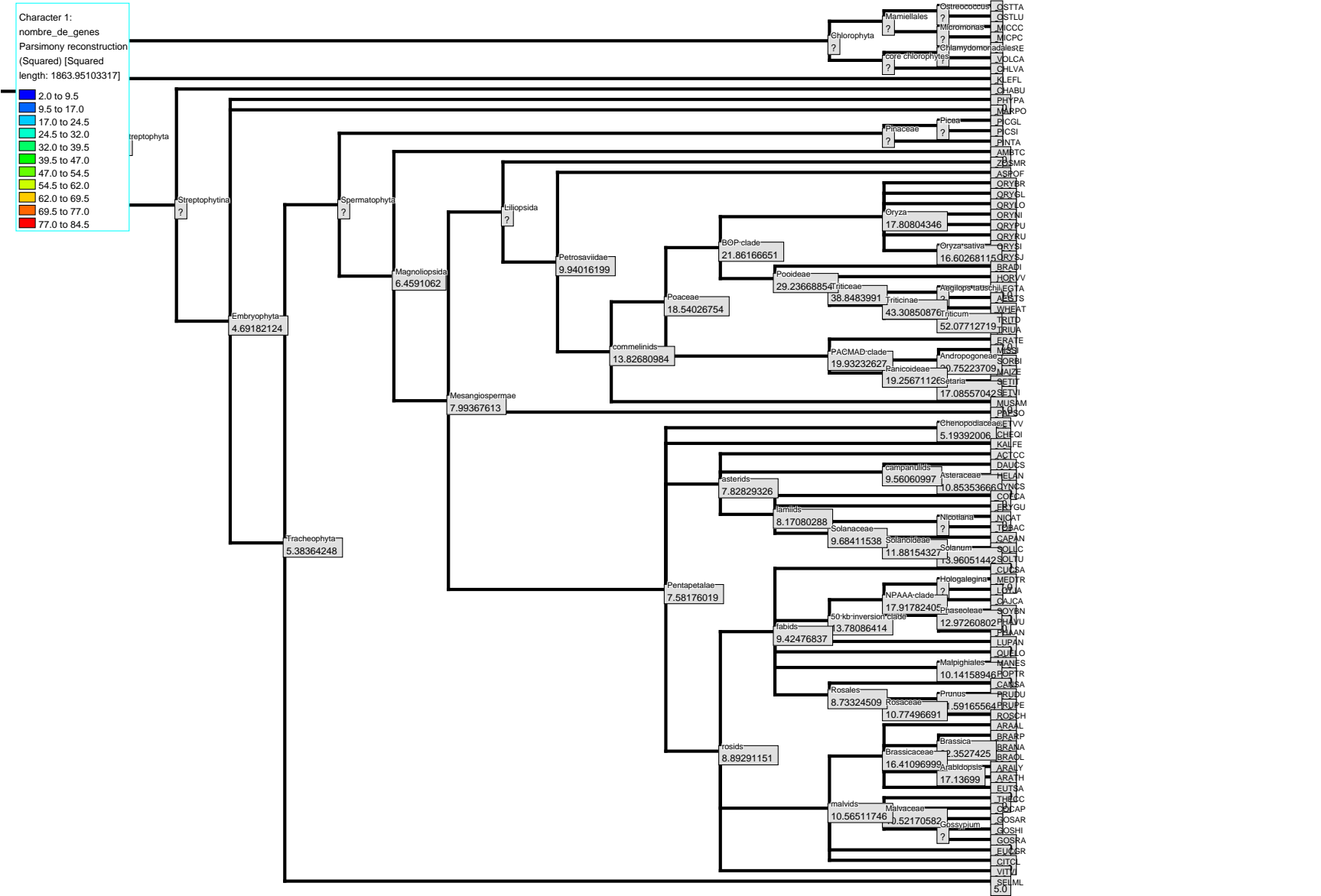

Supplement: S3 File — (PDF) [file pone.0295021.s003.pdf]

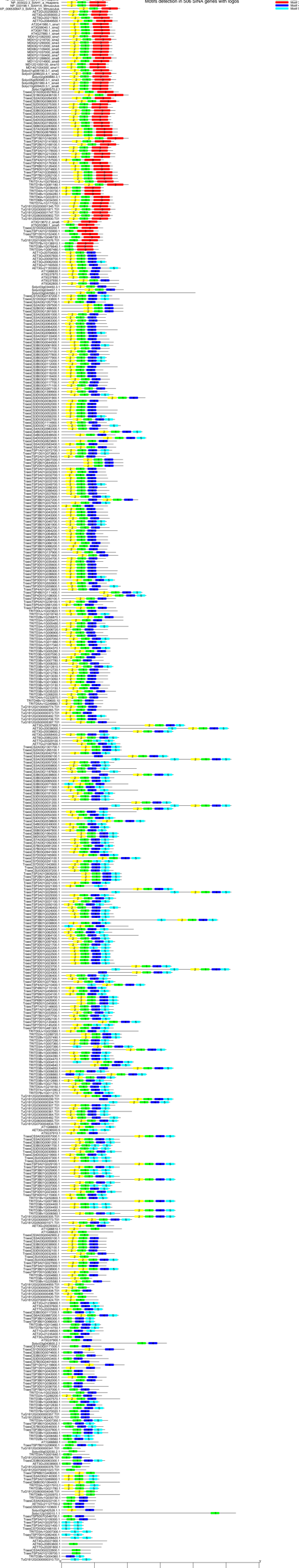





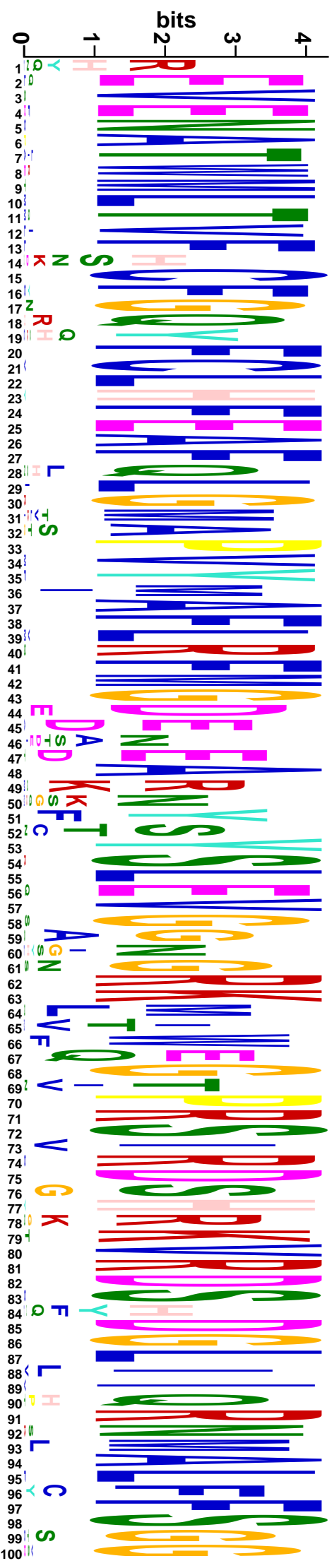

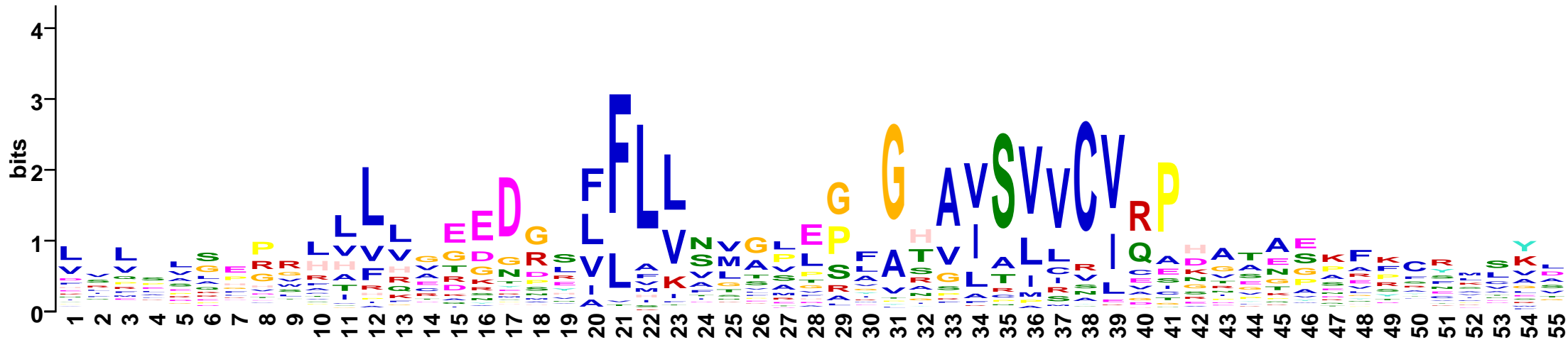

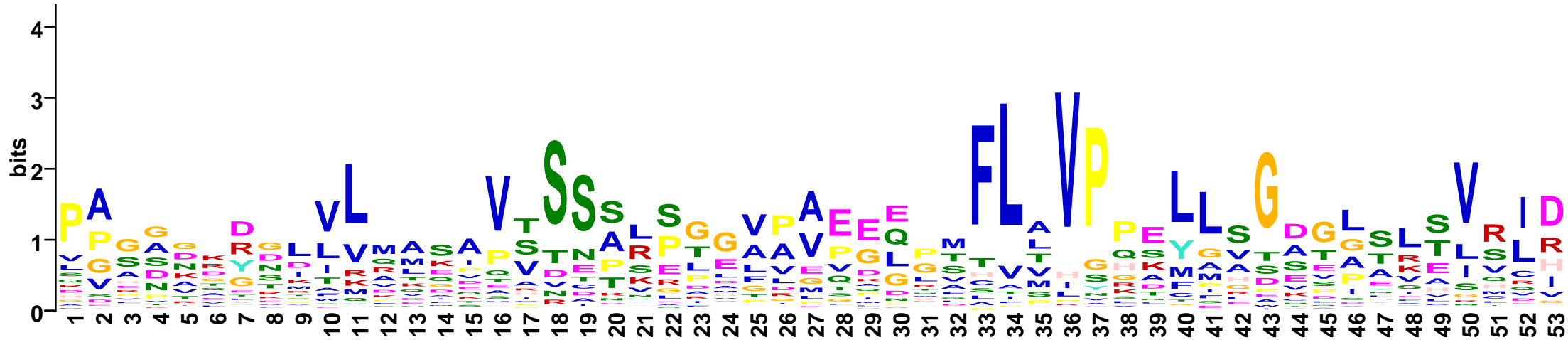

Supplement: S5 File — (PDF) [file pone.0295021.s005.pdf]
